# Evaluation of the whole proteome to design a novel mRNA-based vaccine against multidrug-resistant *Serratia marcescens*

**DOI:** 10.3389/fmicb.2022.960285

**Published:** 2022-10-18

**Authors:** Muhammad Naveed, Muhammad Saad Mughal, Khizra Jabeen, Tariq Aziz, Sumaira Naz, Nausheen Nazir, Muhammad Shahzad, Metab Alharbi, Abdulrahman Alshammari, Satya Sai Sadhu

**Affiliations:** ^1^Department of Biotechnology, Faculty of Science and Technology, University of Central Punjab, Lahore, Pakistan; ^2^Pak-Austria Fachhochschule: Institute of Applied Sciences and Technology, Mang, Pakistan; ^3^Department of Biochemistry, University of Malakand, Chakdara, Pakistan; ^4^Institute of Basic Medical Sciences, Khyber Medical University, Peshawar, Pakistan; ^5^Department of Pharmacology and Toxicology, College of Pharmacy, King Saud University, Riyadh, Saudi Arabia; ^6^Chemistry Department, Northern Michigan University, Marquette, MI, United States

**Keywords:** *Serratia*, gram-negative, epigenetics, immunity, vaccine, TLR-3, linkers, *in vitro*

## Abstract

*Serratia marcescens*, a Gram-negative bacterium, is one of the known disease-causing pathogens. It is resistant to ampicillin, macrolides, cephalosporins, cefotaxime, and ceftazidime. The only antibiotic that has been proven to be effective against *S. marcescens* is gentamicin. By causing epigenetic alterations, bacteria can also become resistant to all antibiotics. Many epigenetically related proteins were studied, and four proteins were selected in this regard for epitope evaluation and their subsequent use in the development of a messenger ribonucleic acid (mRNA) vaccine. A series of immune-informatics tools used to build this mRNA vaccine elicited cellular and humoral immunity. Molecular docking between epitopes and alleles of the major histocompatibility complex (MHC) was performed. The vaccine was developed using 37 epitopes, an adjuvant that is a TLR-4 agonist known as resuscitation-promoting factor E (RpfE), subcellular trafficking structures, secretion boosters, and linkers. This proposed architecture was found to cover 99.6% of the population during testing. During testing, it was proven that it was both effective and safe. To confirm our idea, we performed an *in silico* immunological simulation of vaccination. The codon was also optimized to ensure that the mRNA reached the cytoplasm of a human host and underwent efficient translation. TLR-4 and TLR-3 were also docked against the secondary and tertiary structures of the vaccine peptide. Furthermore, the vaccine's stability was confirmed by molecular dynamics simulation. In summary, this vaccine construct can be a potential candidate against *S. marcescens* and is suitable for *in vitro* analyses to validate its effectiveness.

## Introduction

Antimicrobial resistance (AMR) is a serious problem (Prestinaci et al., 2015). Antibiotic therapies are no longer effective due to the emergence of new resistant strains (Jansen and Anderson, 2018; Micoli et al., 2021), and AMR has severe effects on human health globally, resulting in high levels of illness and mortality as well as severe economic consequences. According to estimates, it results in 10 million deaths per year and a total economic loss of $100 trillion. To successfully combat AMR, it is necessary to develop novel and innovative strategies such as monoclonal antibodies, new diagnostics and medications, new vaccinations targeting antibiotic-resistant bacteria and improved coverage of current vaccines (Micoli et al., 2021). Multidrug resistance has increased globally, which is considered a threat to public health. Several recent investigations have reported the emergence of multidrug-resistant (MDR) bacterial pathogens from different origins, which has increased the need for effective new vaccines. In addition, it has been accomplished to apply antimicrobial susceptibility testing routinely to detect the preferred antibiotic as well as to screen for emerging MDR strains has been achieved.

Although *Serratia marcescens* is a rare species of Gram-negative Enterobacteriaceae, it is now one of the known disease-causing pathogens (Ferreira et al., 2020). This can cause a wide range of infections in immunocompromised patients, especially those in intensive care units (ICUs) and neonatal intensive care units (NICU), including respiratory, blood, skin, urinary, and ocular infections with a high incidence of contact lens-related keratitis, catheter-related infections, sepsis, and meningitis. *Serratia* species is at the top of the World Health Organization (WHO) priority list of MDR bacteria (Carmeli et al., 2018) that pose a severe threat to human health globally (Bloom et al., 2018; Jansen and Anderson, 2018; Alghamdi, 2021). According to recent studies, the number of *S. marcescens* multidrug-resistant strains has increased globally. As a result, innovative and effective treatments as well as prevention techniques are urgently needed (López-Siles et al., 2021).

Many of the strains available are thought to be endemic. There are many antibiotic treatments, but the issue is that bacteria have acquired resistance over time. The only known antibiotic that has shown some potential is gentamicin. Gentamicin is administered *via* injection as it is in the liquid form. The injection is mainly given intravenously (into a vein) or intramuscularly (into a muscle). It has shown some resistance to polymyxin B, nalidixic acid, cephalosporins, and kanamycin (O'neill and nations, 2014). The pathogenicity of *S. marcescens* relies on different virulence factors, including the pore-forming toxin hemolysin, the protease serralysin, or a phospholipase. *S. marcescens* invades and destroys flies' intestinal epithelium, resulting in endothelial cell (EC) death. Bacteria in the midgut cause the local release of adenosine monophosphate (AMP) *via* the immune deficiency (IMD) signaling pathway and are hypothesized to induce local ROS production *via* the DuOx enzyme. Surprisingly, a small but significant number of bacteria can pass through the gut barrier and enter the hemolymph.

Many infectious agents or pathogens can cause changes in epigenetics affecting the host, particularly in immune cells. They cause these changes with different metabolites and byproducts. Deoxyribonucleic acid (DNA) and hydroxy methylation, ribonucleic acid- (RNA-) based modifications, chromatin remodeling, and histone regulation are examples of these alterations. In *S. marcescens*, epigenetic modifications can be observed in the evolution centered on N^6^-methyladenosine (m6A) methylation. Time-evolving strains of *S. marcescens* have polymorphic m6A epiloci (Kim et al., 2015). The m6A methylation targets are GATC motifs, and changes in m6A epiloci are found to have an expressive linkage between gene expression in *S. marcescens* and m6A methylation (Pan et al., 2022). Epigenetic and genetic traits are linked to physical traits. In *S. marcescens*, epigenetic changes with respect to adenine methylation and genetic changes also contribute to phenotypic adaptation and these changes are quite labile (Bruneaux et al., 2021).

**Figure 1:**
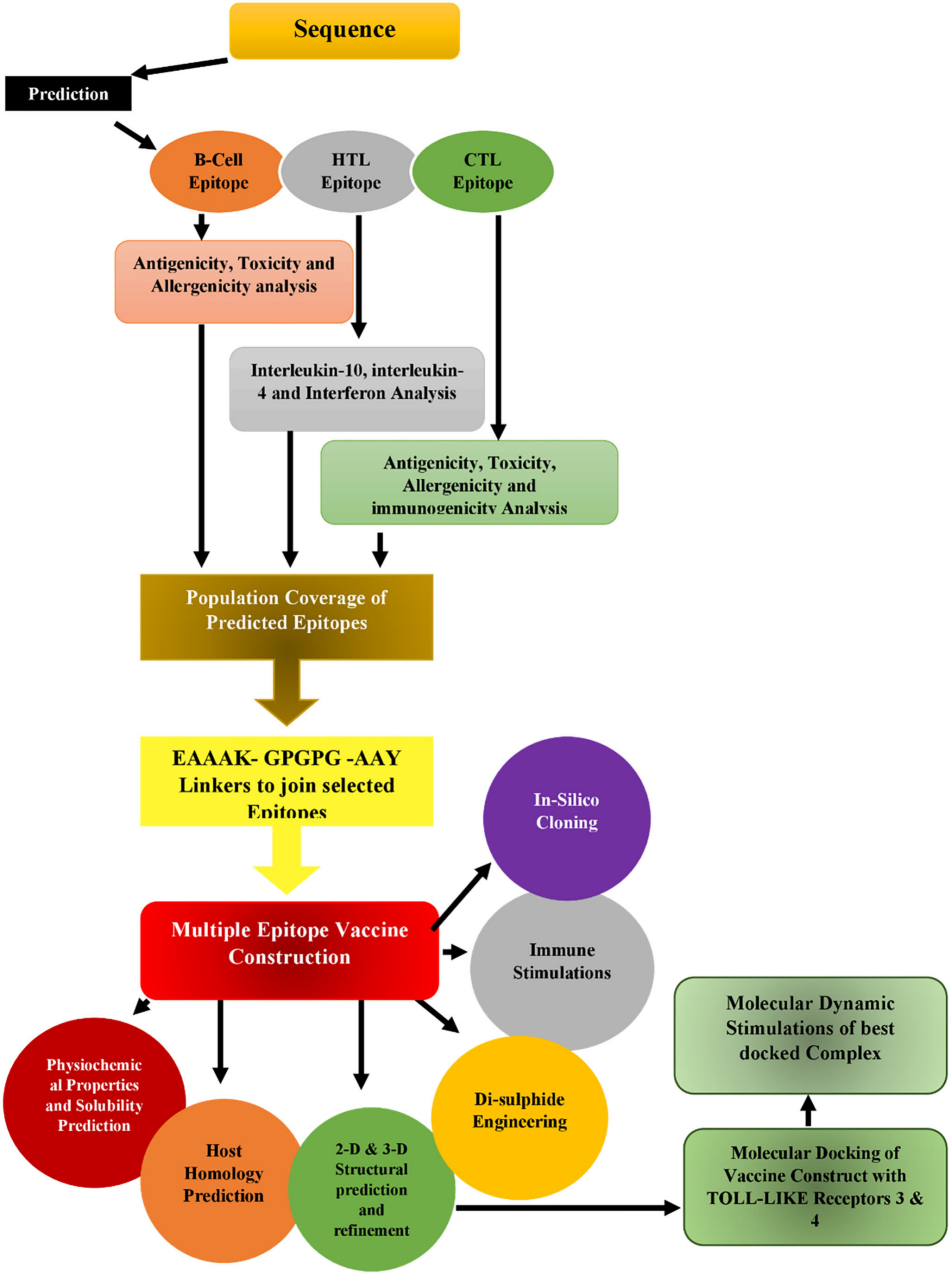
Flow diagram of an *in silico* vaccine construct.

This study mainly focused on the design of a messenger RNA (mRNA) peptide-based vaccine using antigenic proteins from *S. marcescens* using a purified in silico approach, as displayed in Figure 1. Many tools have been used in the process to predict and validate B-cell, cytotoxic T-lymphocyte (CTL), and helper T-lymphocyte (HTL) epitopes of proteins, and they have also been checked for autoimmunity. The vaccine construct is designed by combining all the epitopes with linkers and an adjuvant. RpfE was an adjuvant, and population coverage, antigenicity, allergenicity, and toxicity of vaccine were also accessed. Secondary and tertiary structures have been predicted for the vaccine. Moreover, molecular docking is performed between the vaccine construct and TLR-3 and TLR-4. In conclusion, the stability of the vaccine was also validated by molecular dynamics simulation.

## Methodology

### Retrieval of bacterial protein sequences

Sequences of bacterial proteins involved in epigenetics were obtained from the National Center for Biotechnology Information (NCBI) database at https://www.ncbi.nlm.nih.gov/ and were (1) D-alanyl-D-alanine carboxypeptidase/D-alanyl-Dalanine-endopeptidase (accession number WP_041033700.1), (2) phospholipase C, specific for phosphocholine (accession number WP_141960268.1), (3) spore coat U domain containing protein (accession number- WP_048321499.1), and (4) TonB-dependent receptor (accession number WP_033636744.1) in FASTA format. Of the 10 proteins, 4 proteins are essential proteins, 2 are virulent proteins, 1 is a resistant protein, and 1 protein is an essential and virulent protein.

### Prediction of B-cell epitopes and their estimation

Linear B-cell epitopes were predicted using ABCpred, an online web server. It predicts epitopes using a machine learning algorithm. The proteins that were chosen were submitted with a threshold of 0.51, and epitope lengths were selected at 16 mer. The top seven epitopes were chosen and employed in the development of vaccines (Saha and Raghava, 2006).

### Prediction and estimation of HTL epitopes

The Immune Epitope database (IEDB) major histocompatibility complex-II (MHC-II) server was used to predict HTL epitopes. Sequences were submitted to the server in the format of FASTA, and the epitopes were predicted using the NN-align 2.3 settings. The length of the epitopes was chosen to be 15 mer. Furthermore, it has been shown that the predicted epitopes can release IFN-γ, IL-4, and IL-10 (Jakhar and Gakhar, 2020). For that, the servers used are INF epitope (http://crdd.osdd.net/raghava/ifnepitope/predict.php), IL4pred (https://webs.iiitd.edu.in/raghava/il4pred/predict.php), and IL-10pred (https://webs.iiitd.edu.in/~raghava/il10pred/predict3.php).

### Prediction and estimation of CTL epitope

Epitopes with IC50 values greater than 500 were considered. The IEDB's MHC-I server was used to predict CTL epitopes. Sequences of the chosen protein were submitted, and these epitopes were predicted using ANN 4.0 setting. The entire human HLA reference set was selected, and the epitopes were chosen to be 9 or 10 mer in length. Epitopes with IC50 greater than 500 were used in the vaccine development process (Bhasin and Raghava, 2004).

### Homology of predicted peptides with humans

The predicted peptides were searched to evade all autoimmunity chances through the NCBI BlastP database. All remaining peptides with *E* value > 0.05 were then considered as non-homologous peptides, which were then used in the vaccine construct (Pearson, 2013).

### Epitope estimation for antigenicity, allergenicity, and toxicity

The identified epitopes were all determined to be antigenic, allergenic, and toxic. The VaxiJen online web server was used to predict the antigenicity of the epitopes. For this, the bacteria parameter and a threshold of 0.4 were selected. To predict toxicity, the ToxinPred server was used and default settings were selected (Khan et al., 2022). Using default parameters, AllerTOP was used to determine allergenicity. Only antigenic, non-toxic, and non-allergenic epitopes were retained for continuous vaccination development (Bibi et al., 2021).

### Sequence alignment of variations of proteins

The NCBI database was used to collect all protein variants. The BioEdit 7.2 sequence alignment and analysis software was run for alignment purposes (Stafford et al., 2014). Furthermore, all anticipated epitopes are validated to ensure that they are located in a conserved area.

### Evaluation of binding energies

To evaluate binding affinity, a molecular docking simulation was performed between T-lymphocytes and MHC alleles. The tertiary structure of the MHC alleles was downloaded from the RCSB PDB database (Zardecki et al., 2016). Structures were visualized using the PyMol software, and energy was saved using the Swiss-PDB viewer (Stafford et al., 2014; Yuan et al., 2017). The PEP-FOLD 3.5 server is used to convert epitopes into three dimensional (3D) structures, and energy is minimized using the Swiss-PDB viewer. The Cluspro 2.0 server was used for docking and binding affinity computation (Comeau et al., 2004). PyMol and Discovery Studio are then used for additional visualization and analysis.

### Analysis of population coverage

The IEDB's Coverage of Population web server (http://tools.iedb.org/population/) calculated the coverage for vaccine design-targeted T-lymphocyte epitopes and associated MHCI and MHCII alleles. This calculated value is determined by the coverage of the recognized MHC alleles by the epitopes of the construct. This is due to the fact that MHC allele distribution varies globally among geographical or ethnic groups.

### mRNA vaccine construct design

From the N-terminus to the C-terminus, the mRNA vaccine construct was proposed as follows.

**Figure 2:**
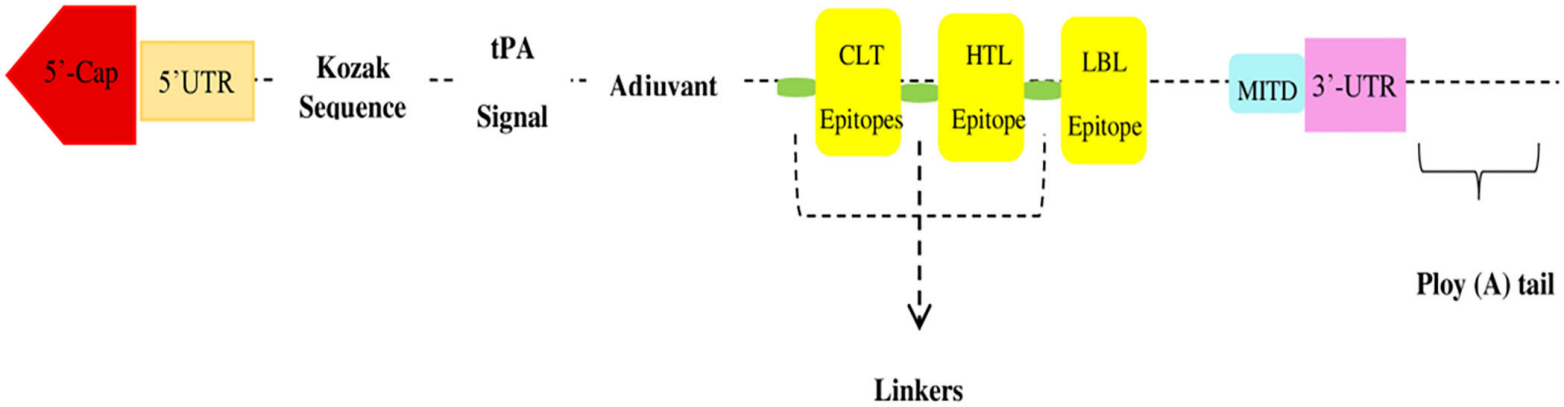
Flow diagram of vaccine construct from N-terminal to C-terminal.

All suggested epitopes were connected using three linkers: AAY, GPGPG, and KK linkers. These linkers allow the epitopes to operate independently, as shown in Figure 2. They are cleavable, flexible, and stiff. To increase the adaptive immune response, an adjuvant RpfE was used. A Kozak sequence must be included in the mRNA vaccine. Furthermore, two structures, tPA (P00750) in the 5′ region of the construct and MITD (Q8WV92), were added to the 3′ locus of the mRNA vaccine.

### Vaccine prediction for physiochemical properties and estimation

The antigenicity of the vaccine design was predicted using VaxiJen 2.0 and ANTIGENpro servers. VaxiJen predicts antigenicity using the vaccine's physiochemical characteristics, whereas the ANTIGENpro server uses a machine learning algorithm to predict outcomes. For allergenicity, AllerTOP server was used and for toxicity, the ToxinPred server was used. The Protpram website was used to calculate physiochemical parameters, which provide vaccination attributes such as composition, molecular weight, predicted pI, and GRAVY (Raza et al., 2022).

### *In silico* immune simulation

C-ImmSim, an online simulation web server (http://150.146.2.1/CIMMSIM/index.php), was used to test immunological simulation during vaccine development. Vaccines are typically administered in two to three doses over a 4-week period. All the settings were set to default, with injections at time steps of 184 and 168.

### Vaccine construct codon optimization

Codon optimization was performed to successfully produce the peptide vaccine construct in human cells. This was accomplished using the GenSmart Codon Optimization program (http://www.genscript.com/). GenScript utilized rare codon analysis tools for quality assessment (http://www.genscript.com/) (GS). The effectiveness of mRNA expression translation (CAI) was measured by the Codon Adaptation Index.

### mRNA vaccine secondary structure prediction

The RNAfold tool (http://rna.tbi.univie.ac.at/cgi-bin/RNAWebSuite/RNAfold.cgi) of ViennaRNA Package 2.0 was used to determine the secondary structure of the mRNA vaccine. This method was also used to get the free energy minimum (MFE). The RNAfold tool used McCaskill's method to calculate the secondary structure.

### Prediction and validation of vaccine secondary and 3D structures

PSIPRED and Robetta servers were used to predict the secondary and tertiary structures, respectively. The accuracy was 84.2% according to the PSIPRED server (http://bioinf.cs.ucl.ac.uk/psipred/). The Robetta server (https://robetta.bakerlab.org/) predicted five different 3D structures, which were then evaluated using PROCHECK, ERRAT (https://saves.mbi.ucla.edu/), and the ProSA-web server (https://prosa.services.came.sbg.ac.at/prosa.php).

### Prediction of discontinuous B-cell epitopes

The Ellipro online web server (http://tools.iedb.org/ellipro/) was used to predict discontinuous B-cell epitopes in the protein structure. It utilized the geometrical properties of the 3D model and achieved the greatest area under the curve (AUC) value of 0.732 for any protein model (Wang et al., 2016).

### Vaccine docking with TLR-3 and TLR-4

The ClusPro server was utilized to dock the vaccine with TLR-3 (PDB ID: 1ZIW) and TLR-4 (PDB ID: 3FXI). The PRODIGY tool on the HADDOCK server (https://haddock.science.uu.nl/) was used to determine the binding free energy (G), dissociation constant (Kd), and the percentages of charged and polar amino acids identified on the receptor–ligand 3D non-interacting surface. Moreover, to analyze and visualize the receptor and ligand interaction, the PDBsum web server was used (Laskowski et al., 2018).

### Molecular dynamics simulation

The iMOD server was used for dynamic simulation analysis of the TLR-3- and TLR-4-vacine complexes (http://imods.Chaconlab.org/), confirming the stability and motion of atoms and molecules in the vaccine.

## Results

### B-cell epitopes and their estimation

From each included protein, we chose the top epitopes predicted by the ABCpred website, as presented in Table 1. Only epitopes with antigenic, non-toxic, non-allergenic, and non-homologous properties for vaccine construction were selected. Moreover, all screened epitopes were analyzed if they were homologous to Homo sapiens, to eliminate from vaccine design, which might introduce autoimmunity. In Table 1, we chose 12 B-cell epitopes initiating target proteins for inclusion in the vaccine design.

**Table 1:** List of epitope candidates to design vaccines.

**Cell type**	**Sequence of epitopes**
CD8+ Cytotoxic T-Lymphocyte	WLLPAILAL
	LEDRLVETL
	QTPFGAGWSW
	FQFALANAF
	FTIPLPGDR
	SAADVAVVV
	SSNVNFPLY
	CLIGTAPNV
	TTNGTVTLP
	QTYGAKIAR
	SEYVWNYEL
	RYFHGTQDEF
CD4+ helper T-Lymphocyte	WLLPAILALAGCSSS
	GARAGAPGRVSFYPA
	PGARAGAPGRVSFYP
	AFAAPISALNYAFTP
	EKRFQVHEPNISAWR
	AGFRQYRAASIQVGN
	HELPFQFALANAFTL
	SLNLLSLILISSNVN
	LSLILISSNVNFPLY
	NVGANAFLSGTRPRL
	NAFLSGTRPRLNLSL
	ARAPAYTANMGAKYQ
	AKYQFLKGWELSSNV
	TGEQRGDTL
B-Lymphocyte	SGDPTLHPDDL
	GRKTQGKGD
	QREVYSHRTTPRM
	SSQRINTRTLGLRLDS
	MAVANTDGSGD
	TTVWDSTNKQSGAGT
	QPEVRLRPTG
	FAAQRHESVGN
	AETKSNETYQD
	DRQRRRSEADL
	RLEREHRRRDG

### Prediction and estimation of CTL epitopes

Among the targeted four proteins, we chose CTL epitopes from the MHC-I IEDB database. The method used for the prediction of epitopes was Artificial Neural Network (ANN) and the epitopes having ic50 range up to 500 was selected. Furthermore, we exclusively chose antigenic, allergenic, non-toxic, and non-homologous epitopes. Consequently, as shown in Table 1, seven epitopes were chosen for vaccine construction.

### Prediction and estimation of HTL epitopes

Outer membrane proteins were evaluated to identify potential HTL epitopes. Only the epitopes studied that were antigenic, non-toxic, non-allergenic, and lastly non-homologous and capable of inducing IL-4, INF-γ, and IL-10, were selected. As shown in Table 1, 16 potential cytokine-triggering epitopes in a conserved area were chosen.

### Molecular docking interaction of epitopes and MHC alleles

**Figure 3:**
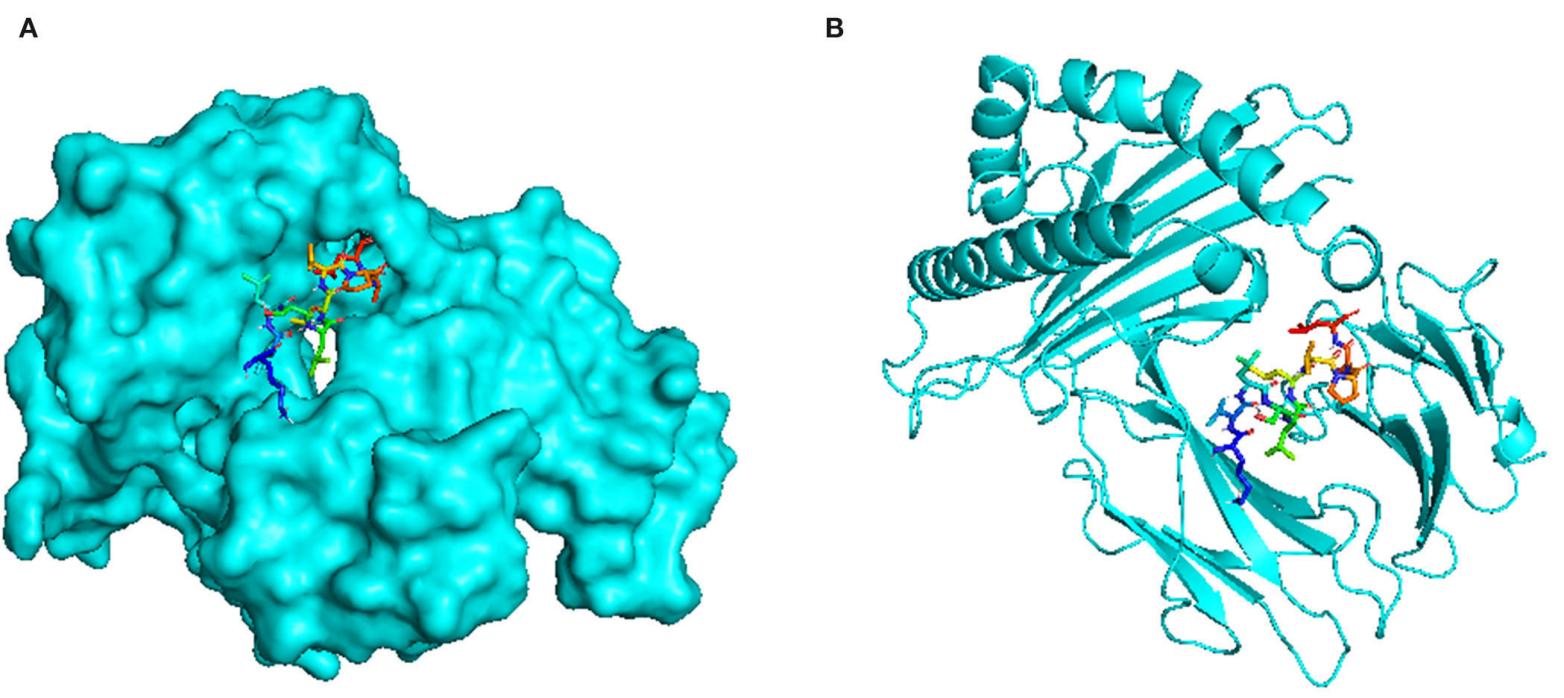
Docking visualization between the epitope AGFRQYRAASIQVGN and its corresponding MHC allele (HLA-DRB1*01:01) using the PyMol software: **(A)** Surface view **(B)** Cartoon view.

**Figure 4:**
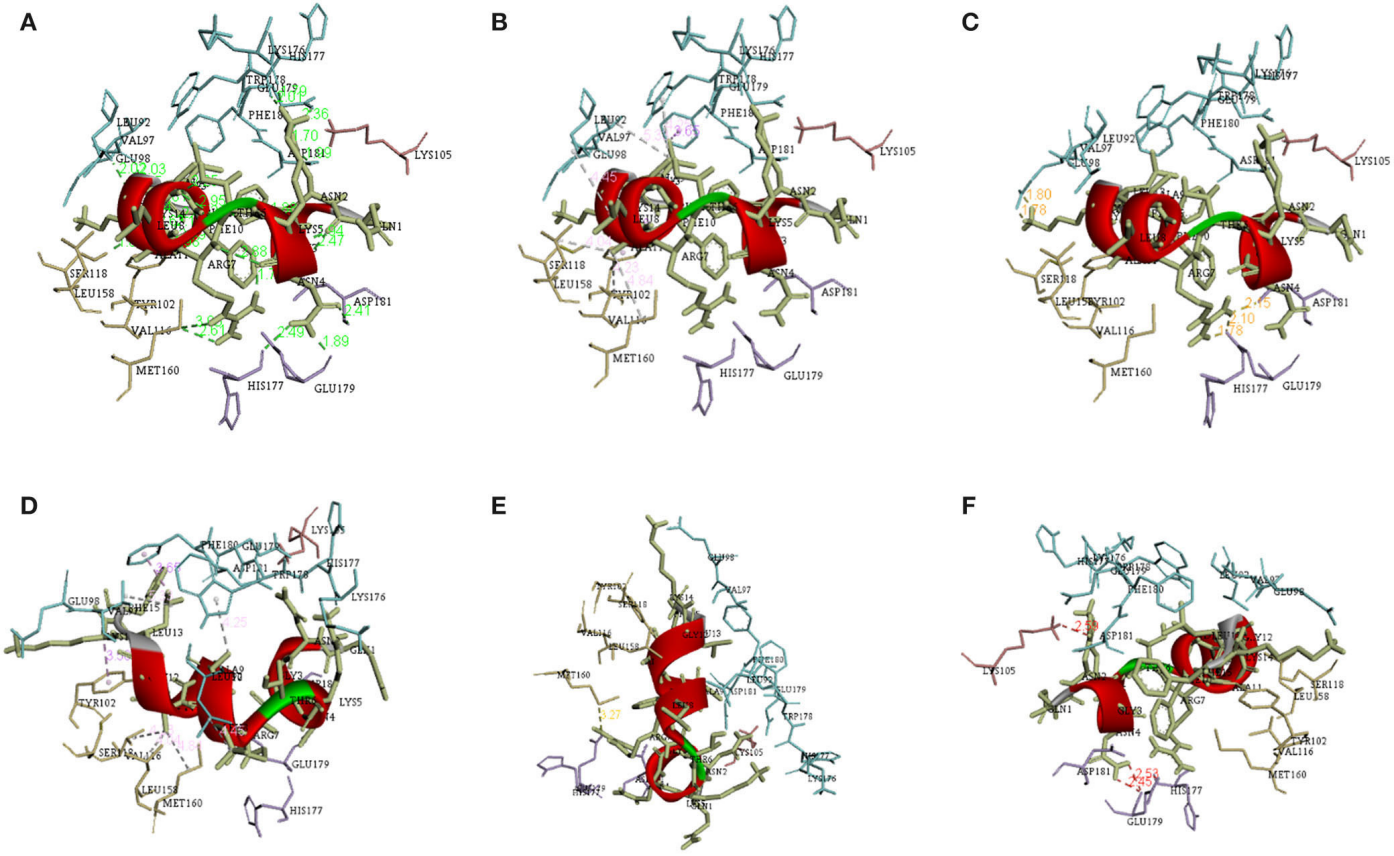
Different interactions between the epitope and its corresponding MHC allele visualized using the discovery studio. **(A)** Conventional hydrogen bonds **(B)** Salt bridge, attractive charge interactions **(C)** Hydrophobic interactions **(D)** Cation-Pi interactions **(E)** Donor-Donor clash **(F)** Pi Donor hydrogen bond.

There are a total of 23 T-lymphocyte epitopes, with 131 MHC alleles overall. Some epitopes recognize just one MHC allele, whereas others detect numerous alleles, such as an epitope can attach up to 18 MHC alleles, as shown in Supplementary Table 1. We have chosen five epitopes with corresponding MHC alleles from among them for molecular docking investigation. In Table 2, docking outcomes from the server of ClusPro 2.0 in the form of energy affinity has been demonstrated. The AGFRQYRAASIQVGN epitope with the corresponding MHC alleles (HLA-DRB1^*^01:01) shows the strongest binding affinity of −8.502 kcal/mol. Consequently, epitopes are competently bound to the MHC allele binding fork, as shown in Figure 3. Furthermore, all possible interactions of the targeted epitope and residues of alleles were evaluated as shown in Figure 4.

**Table 2:** Molecular docking of T-lymphocyte epitopes with their corresponding MHC alleles and their binding affinity.

**Types of epitope**	**Epitopes**	**MHC alleles**	**PDB ID**	**Binding affinity**
CTL	SSNVNFPLY	HLA-B*35:01	4PR5	−8.1
	QTYGAKIAR	HLA-A*03:01	3RL1	−7.9
	RYFHGTQDEF	HLA-B*15:01	1XR8	−6.8
HTL	AGFRQYRAASIQVGN	HLA-DRB1*01:01	2FSE	−8.7
	ARAPAYTANMGAKYQ	HLA-DRB1*15:01	1BX2	−7.5

### Vaccine construct design

The proposed design of the vaccine construct is as follows.

5′ m7GCap- 5′ UTR–Kozak sequence-EAAAK-linker-adjuvant-MKNARTTLIAAAIAGTLVTTSPAGIANADDAGLDPNAAAGPDAVGFDPNLPPAPDAAPVDTPPAPEDAGFDPNLPPPLAPDFLSPPAEEAPPVPVAYSVNWDAIAQCESGGNWSINTGNGYYGGLRFTAGTWRANGGSGSAANASREEQIRVAENVLRSQGIRAWPVCGRRGGPGPGWLLPAILALAGCSSSGPGPGGARAGAPGRVSFYPAGPGPGPGARAGAPGRVSFYPGPGPGAFAAPISALNYAFTPGPGPGEKRFQVHEPNISAWRGPGPGAGFRQYRAASIQVGNGPGPGHELPFQFALANAFTLGPGPGSLNLLSLILISSNVNGPGPGLSLILISSNVNFPLYGPGPGNVGANAFLSGTRPRLGPGPGNAFLSGTRPRLNLSLGPGPGARAPAYTANMGAKYQGPGPGAKYQFLKGWELSSNVKKTGEQRGDTLKKSGDPTLHPDDLKKGRKTQGKGDKKQREVYSHRTTPRMKKSSQRINTRTLGLRLDSKKMAVANTDGSGDKKTTVWDSTNKQSGAGTKKQPEVRLRPTGKKFAAQRHESVGNKKAETKSNETYQDKKDRQRRRSEADLKKRLEREHRRRDGAAYWLLPAILALAAYLEDRLVETLAAYQTPFGAGWSWAAYFQFALANAFAAYFTIPLPGDRAAYSAADVAVVVAAYSSNVNFPLYAAYCLIGTAPNVAAYTTNGTVTLPAAYQTYGAKIARAAYSEYVWNYELAARYFHGTQDEF-AAY-MITD sequence–Stop codon−3′ UTR–Poly (A) tail.

### Evaluation of the physicochemical properties of the vaccine for

VaxiJen, ANTIGENpro, the AllerTOP, ToxinPred, and SolPro tools were used to evaluate the antigenicity, allergenicity, toxicity, and solubility of the construct. The vaccine was found out to be antigenic, non-allergenic, non-toxic, and soluble. Moreover, the ExPasy ProtParam service was used to determine the physiochemical profile of the construct as presented in Supplementary Table 2. All the physiochemical properties of the vaccine construct predict that the construct is thermally stable. The GRAVY was found to be −0.419, indicating that the vaccine is hydrophilic. This proved that the mRNA vaccine design could be a possible vaccine candidate based on these findings.

### Population coverage prediction

The IEDB population coverage tool predicted global population coverage by combining MHC-I and MHC-II, totaling 65 alleles of corresponding 23 epitopes. Finally, the global vaccine coverage rate would be around 99.6%.

### Immune simulation response

**Figure 5:**
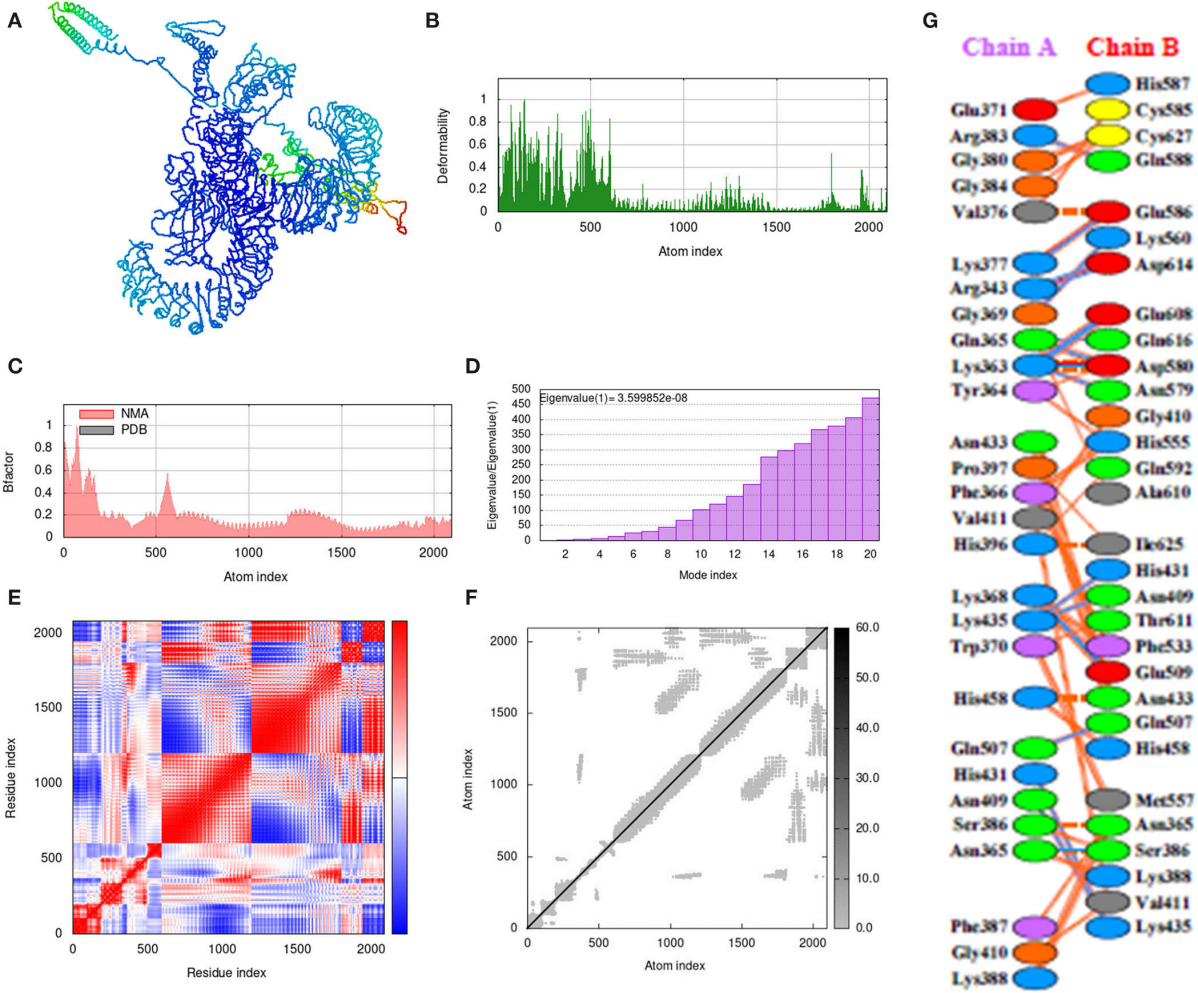
The ElliPro server of the Immune Epitope database (IEDB) for the prediction of 11 conformational B-cell epitopes: (I) Position of conformational B-cell epitope 2D illustration. (II) 3D models of B-cell epitopes where yellow spheres present conformational B-cell epitopes. **(A)** 35 residues with a score of 0.819, **(B)** 155 residues with a score of 0.819, **(C)** 42 residues with a score of 0.802, **(D)** 10 residues with a score of 0.675, **(E)** 13 residues with a score of 0.634, **(F)** 51 residues with a score of 0.615, **(G)** 9 residues with a score of 0.612, and **(H)** 11 residues with a score of 0.579.

To initiate the immunological response, we employed three vaccination doses (Figure 5). The second and third responses outperformed the first response. Immunoglobulin levels were high after antigen suppression, and IgM was found to be created in greater quantities compared to IgG. This rise may indicate that immunological memory has developed as a result of antigen exposure. The presence of B-cell isotypes over an extended period of time demonstrates the formation of memory in the B-cell population. Furthermore, the development of memory also leads to an increase in CTL and HTL cells. Additionally, there was an increase in macrophage activity although dendritic cell activity remained constant. In addition, IFN-γ and IL-2 levels increased. Innate immunity and the number of epithelial cells, which are part of innate immunity, increases.

### mRNA vaccine prediction for the secondary structure

**Figure 6:**
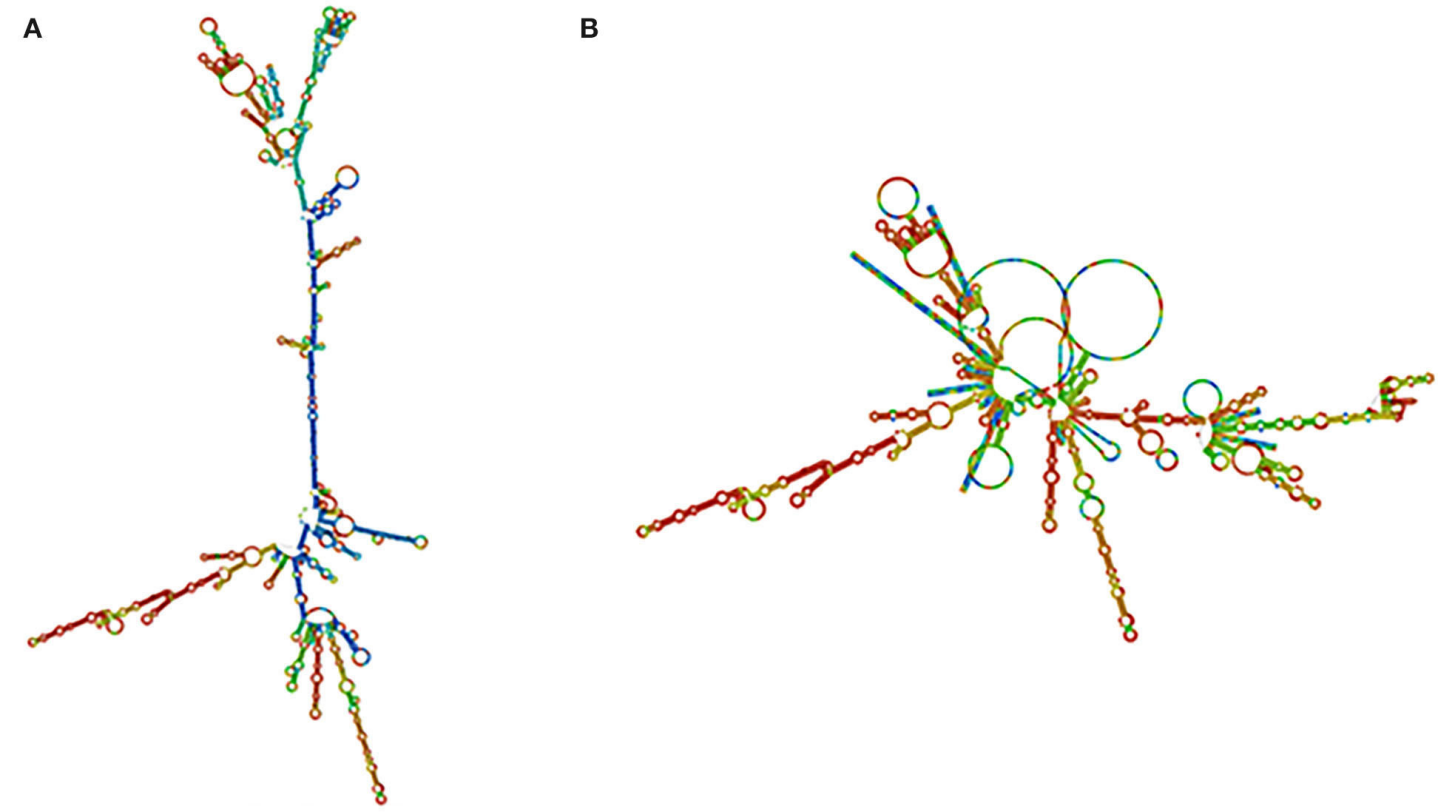
mRNA structure prediction. **(A)** Optimal secondary structure. **(B)** Centroid secondary structure of the vaccine mRNA retrieved using RNA-fold web server.

The RNA fold web server was used to infer the structure of the mRNA vaccine. This server was also used to calculate the structure free energies. We used the vaccine optimized codons as an input. We found out that mRNA vaccine was produced with a minimum free energy of −948.30 kcal/mol, and secondary centroid structure energy was −635.06 kcal/mol as displayed. It has been shown that the mRNA structure will be stable (Figure 6).

### Secondary and tertiary structures of the mRNA vaccine

**Figure 7:**
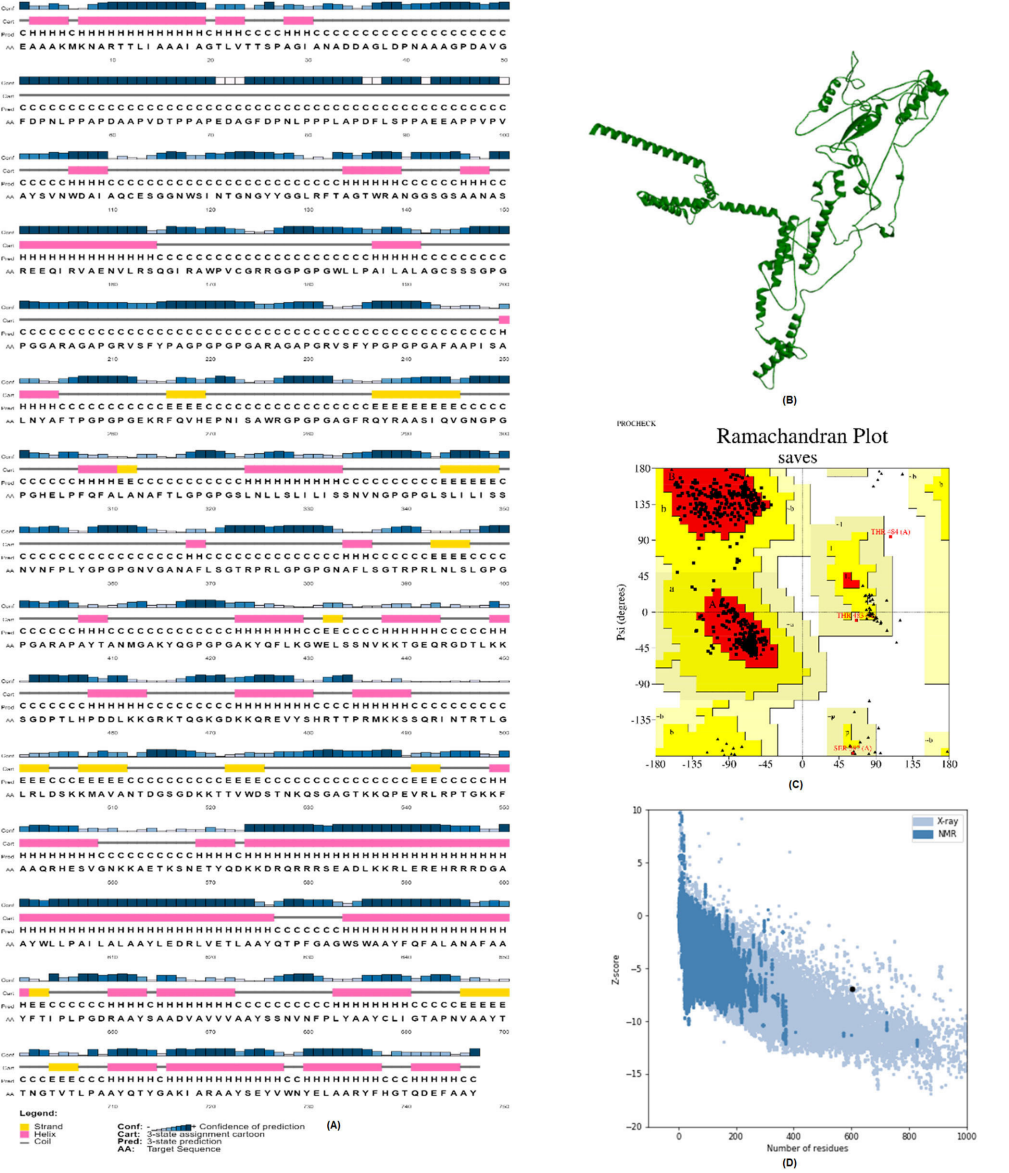
Structure prediction and validation of vaccine construct. **(A)** PSIPRED server results of secondary structure of vaccine. **(B)** The Robetta server used to predict the Tertiary structure of vaccine. **(C)** The PROCHECK server used to analyze the Ramachandran Plot. **(D)** Z-score analyzed by the Pro-SA web server.

We employed the PSIPRED web server to anticipate the vaccine's secondary structure. The structure is mainly made up of alpha helices (Figure 7A). We also used the Robetta server to determine the tertiary structure of the vaccine (Figure 7B). The stereochemical correctness of the structure was then verified using the PROCHECK service. The Ramachandran plot in Figure 7C demonstrates that 96.2% of the residues are in the most preferred zones, 3.3% in the extra allowable zone, and 0.3% in the wide allowed zones. The overall quality factor of the vaccine is 91.5447. The ProSA-web server anticipated a negative *Z*-score of −5.69 for the tertiary protein model, suggesting that it is very consistent.

### Conformational B-cell epitope prediction

**Figure 8:**
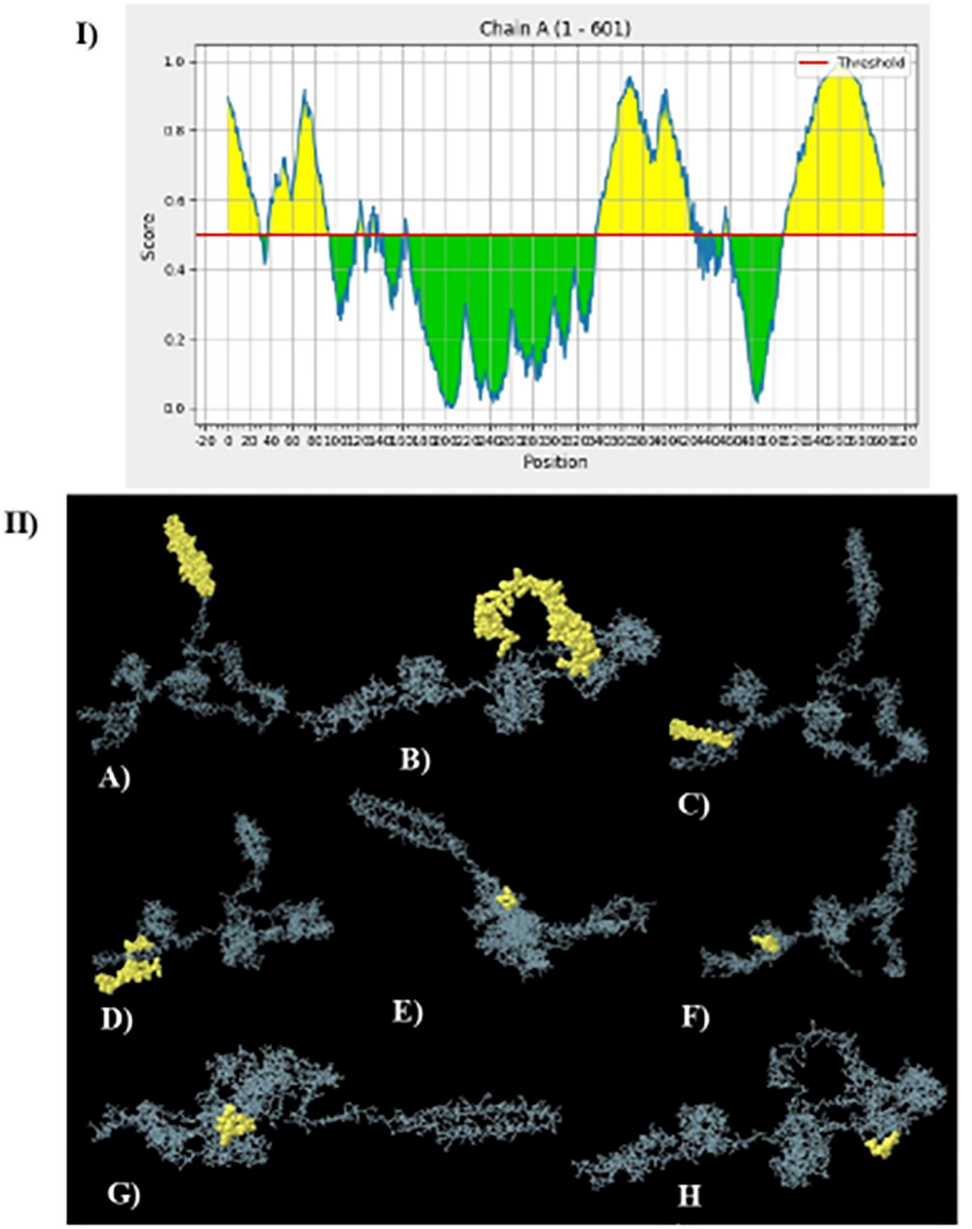
The ElliPro server of IEBD database for the prediction of eleven conformational B-cell epitopes: **(I)** Position of conformational B-cell epitopes 2D illustration. **(II)** 3D models of B-cells epitopes where yellow spheres present the conformational B-cell epitopes. **(A)** 35 residues with a score of 0.819 **(B)** 155 residues with a score of 0.819 **(C)** 42 residues with a score of 0.802 **(D)** 10 residues with a score of 0.675 **(E)** 13 residues with a score of 0.634 **(F)** 51 residues with a score of 0.615 **(G)** 9 residues with a score of 0.612 **(H)** 11 residues with a score of 0.579.

The server ElliPro was used to fold the vaccine model to identify B-cell conformational epitopes. This tool was performed and predicted 11 discontinuous conformational B-cell epitopes. Figure 8 shows the secondary or tertiary models of conformational cell epitopes and a total of 377 residues with predicted scores ranging from 0.577 to 0.848.

ClusPro software was used for molecular docking and confirmation of the putative construct associations with TLR-4 and TLR-3 receptors. Furthermore, we evaluated the most clustered member of each complex for its binding affinities and dissociation constant at 37°C independently using the PRODIGY web server. We used the docked adjuvant complex as a control.

For the TLR-3-vaccine complex, the binding affinity was −14.8 kcal/mol^−1^, while for the control it was −8.1 kcal/mol^−1^. The complex dissociation constant at 25°C for vaccine-TLR-3, it was 1.4E-11 as compared to the control, which had a 1.1E-06 value. In case of the TLR-4-vaccine complex, the binding affinity was −13.9 kcal/mol^−1^ while for the control the binding affinity was −9.0 kcal/mol^−1^. The complex dissociation constant at 25°C for vaccine-TLR-4 was 6.7E-11 as compared to the control, which had a 2.5E-07 value. PDBsum, which is a web server that provides structural information of the entries in the PDB file, is used to evaluate the strongest interaction of vaccines with TLR-3 and TLR-4 receptors.

### Molecular dynamics simulation

**Figure 9:**
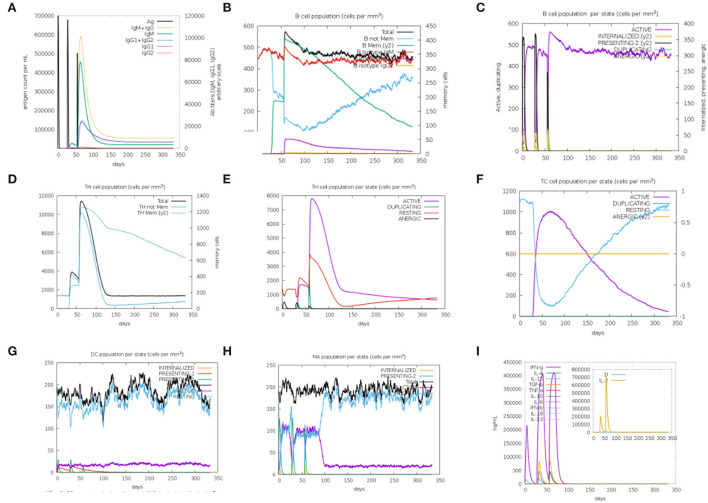
Molecular dynamics simulation, normal mode analysis (NMA), and receptor–ligand interactions: **(A)** vaccine-TLR-3 docked complex using the Cluspro server, **(B)** deformability graph, **(C)** eigenvalue of the vaccine-TLR-3 complex, **(D)** B-factor graph, **(E)** covariance matrix, **(F)** elastic network model using the iMOD server, and **(G)** receptor–ligand interaction using the PDBsum web server.

**Figure 10:**
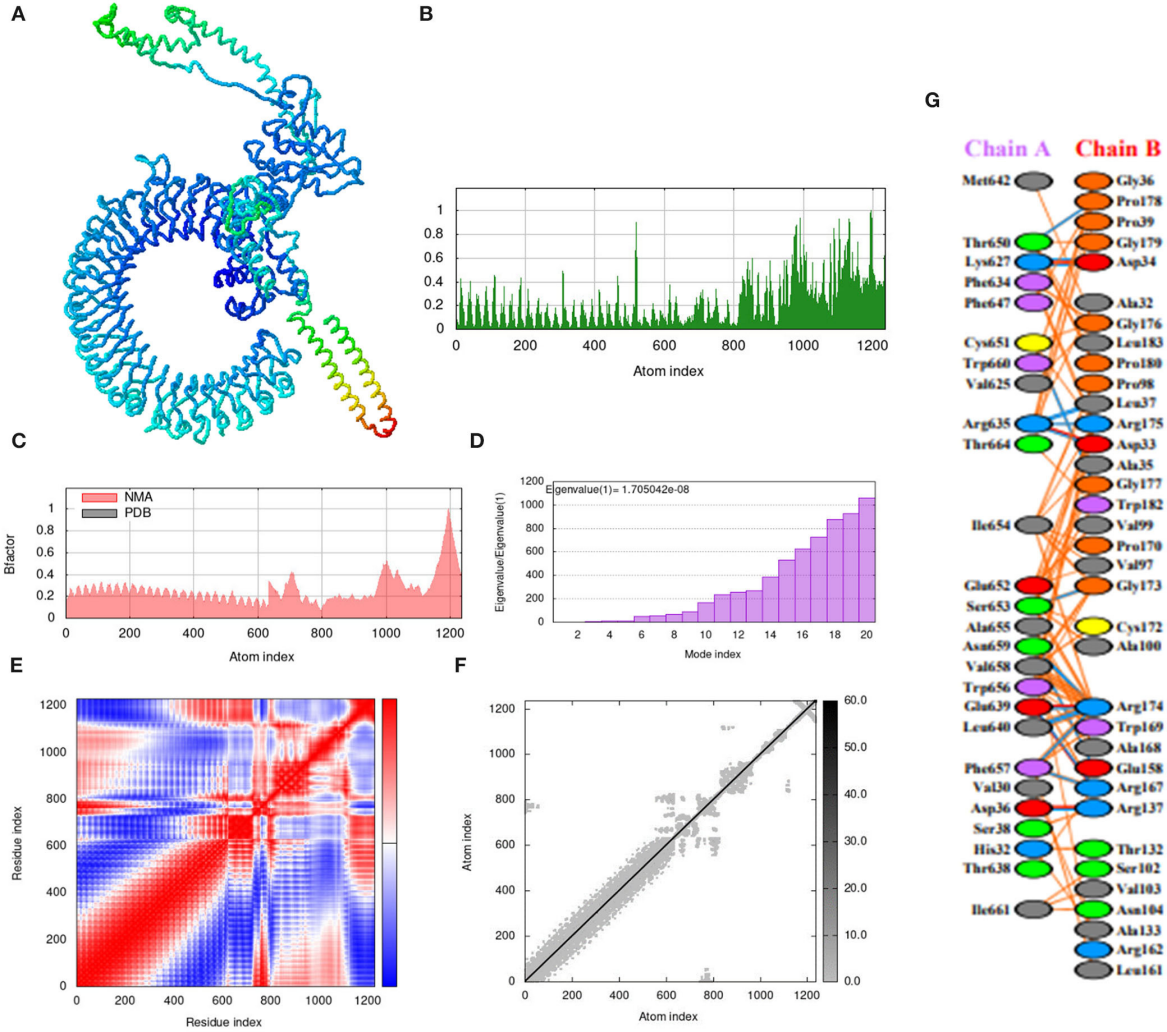
Molecular dynamics simulation, NMA, and receptor–ligand interactions: **(A)** vaccine-TLR-4 docked complex using the Cluspro server, **(B)** deformability graph, **(C)** eigenvalue of the vaccine-TLR-4 complex, **(D)** B-factor graph, **(E)** covariance matrix, **(F)** elastic network model using the iMOD server, **(G)** receptor–ligand interaction using the PDBsum web server.

The iMOD server was used for the molecular dynamic simulation analysis when the Vaccine-TLR3 and Vaccine-TLR4 complexes are exposed to the server as shows in Figures 9A, 10A. Peaks in the deformability graph represent the vaccine deformable loci and show the amino acids that have coiled forms as display in Figures 9B, 10B. NMA (normal mode analysis), which is a computer method for analyzing the flexibility of protein. In Figures 9C, 10C shows, the eigenvalues of both docked complexes. The B-factor graph depicts the complex's link between the Normal Mode Analysis and PDB regions as shows in Figures 9D, 10D. A covariance matrix depicts the relationships among amino acid duplets in the dynamical area as shows in Figures 9E, 10E, where red part indicates correlated residues, the white part for anti-correlated residues and the blue part represents the non-correlated residues and Figures 9F, 10F, represents the elastic network model. Lastly, the receptors and ligands interaction of both docked complexes demonstrated in the Figures 9G, 10G.

## Discussion

*S. marcescens* has now become one of the pathogens known to cause many infections, especially in individuals whose immune system is already compromised. It is believed to be naturally resistant to ampicillin, macrolides, cephalosporins, cefotaxime, and ceftazidime. Many antibiotics are known to be on the market, but the rate of resistance of *S. marcescens* against antibiotics is very high. Only a few antibiotics, including gentamicin, work against it, but the issue of resistance does not make antibiotics a viable solution to bacterial and infectious diseases. Vaccines against these diseases can be helpful and a possible solution, but this is an expensive and time-consuming method (Mugunthan and Harish, 2021). The immune-informatics approach, as compared to the *in vitro* approach, is a gemstone for developing new therapeutics and vaccines. mRNA vaccines have been used to prevent HIV-1, Zika, rabies, influenza viruses, and many other diseases since 1990. Their use is safe and effective, but the only disadvantage of using mRNAs is their degradation by RNases that are part of innate immunity. However, vaccines against rickettsia and *Escherichia coli* diseases, as well as vaccines against several others diseases in late-stage clinical trials, such as *Candida albicans* and many others, are among the successes of the application of immune-informatics methods (Al Tbeishat, 2022).

Epigenetic modifications are related to physical traits, and these changes allow bacteria to survive in the environment. In the case of *S. marcescens*, several *in vitro* studies have demonstrated that its evolution causes not only epigenetic modifications but also modifications in genetic and physical traits. Epigenetic modifications can be observed during evolution, mainly focusing on m6A methylation. The strains of *S. marcescens* have evolved over time with polymorphic m6A epiloci. It turns out that changes in the m6A epiloci have an expressive linkage between the physical traits of *S. marcescens* and its gene expression (Marques-Pereira et al., 2020).

The basic purpose of vaccines is to create a long-lasting memory. As a result, the host will respond effectively and quickly whenever the infectious agent attacks the body again in the future. Moreover, it is essential to predict epitopes that can trigger both B and T cells accurately and incorporate them into the vaccine construction. The HTL epitopes to be selected must be capable of producing IFN-γ, IL-4, and IL-10 cytokines, which play key functions against pathogens (Desai et al., 2020). All immune cells die once the infections is eradicated, apart from memory cells. B-cell receptors (membrane-bound immunoglobulins) recognize epitopes that are processed by these cells *via* MHC-II, which are presented to T cells. As a result, the T-cell receptor (TCR) identified its characteristics. Furthermore, B cells differentiate into memory cells and plasma cells that produce antibodies (Naveed et al., 2021).

The mRNA vaccine has many features that enhance translation and stability. It consists of 5′ methylguanosine (m7G)-cap sequences, poly (A) tails with a length of 120 to 150 bps, globin 5′ and 3′ untranslated regions (UTRs) bordering the mRNA ORF, the Kozak sequence, and a stop codon. Furthermore, the tPA secretory signal sequence and the MITD sequence were introduced to drive it to the endoplasmic reticulum to increase the effectiveness and allocation of the construct. Once in the cytoplasm, the mRNA enters the translation machinery and undergoes post-translational changes, resulting in an immunological response. The ability of the mRNA to produce MHC-I presentation and cytotoxic T-lymphocyte responses is a key advantage of its use as an antigenic source. This allows a high degree of flexibility in the type and amount of antigenic determinants, enabling quick vaccine development. The key aims of mRNA vaccines are their generic production techniques, simplicity of design, and their relative safety.

This study mainly focused on the design of mRNA peptide-based vaccines using antigenic proteins of *S. marcescens* by a pure *in silico* approach. The recommended mRNA vaccine was evaluated to be stable, thermostable, antigenic, non-allergenic, and hydrophilic using immune-informatics approaches. Using molecular simulation, it was found that the vaccine, once administered with three injections, was able to elicit an immunological response as well as to generate memory cells upon exposure and to produce chemokines that promote B-cell and humoral responses. The generation of memory cells was indicated by macrophages, dendritic cells, and the Simson index. Ultimately, the manufactured vaccine is a possible candidate against *S. marcescens* infection.

## Conclusion

In this study, the vaccine construct exhibits favorable physicochemical features as well as immune responses against *S. marcescens*. Proteins involved in epigenetics are shortlisted, and different epitopes, such as those of CTL, HTL, and B cells, are predicted. The vaccine was created using epitopes of CD8+ CTL, CD4+ HTL, and B cells. It has been determined that this vaccination covers 99.6% of the population and will trigger an immune response against *S. marcescens* in the host using various immune-informatics tools or methodologies. The immune response, according to immune stimulation, induced by the vaccine is consistent with our hypothesis. As a result, it is recommended that this vaccine can be used against all evolutionarily altered strains of *S. marcescens* and can be used as a candidate for *in vitro* and *in vivo* research against *S. marcescens*, with numerous serological assays to trigger the response.

## Data availability statement

The original contributions presented in the study are included in the article/Supplementary material, further inquiries can be directed to the corresponding author/s.

## Author contributions

MN, MSM, KJ, and TA: conceptualization and investigation. MN: data curation. MS, MA, and AA: formal analysis. AA, SN, NN, and MA: methodology. MN and TA: resources. MA, SS, AA, SN, and NN: software. MS and TA: supervision. MA, MS, and SS: validation. TA: visualization. MN, TA, and MS: writing—original draft. MSM, KJ, and TA: writing—review and editing. All authors contributed to the article and approved the submitted version.

## Funding

Supporting Project number (RSP2022R462), King Saud University, Riyadh, Saudi Arabia.

## Conflict of interest

The authors declare that the research was conducted in the absence of any commercial or financial relationships that could be construed as a potential conflict of interest.

## Publisher's note

All claims expressed in this article are solely those of the authors and do not necessarily represent those of their affiliated organizations, or those of the publisher, the editors and the reviewers. Any product that may be evaluated in this article, or claim that may be made by its manufacturer, is not guaranteed or endorsed by the publisher.
