# Supplementary material for: Evaluation of the whole proteome to design a novel mRNA-based vaccine against multidrug-resistant Serratia marcescens
Source: Front Microbiol. 2022 Oct 18;13:960285. doi: 10.3389/fmicb.2022.960285 (PMC9624125; doi:10.3389/fmicb.2022.960285)
Supplement: Supplementary file 1 [file Data_Sheet_1.docx]

Table S1: T-Lymphocytes and their corresponding alleles

| **Protein** | **CTL Epitopes** | **MHC I Binding Alleles** | **HTL Epitopes** | **MHC II Binding Alleles** |
| --- | --- | --- | --- | --- |
| **1** | WLLPAILAL | HLA-A*02:06, HLA-A*02:01, HLA-A*02:03, HLA-B*08:01 | WLLPAILALAGCSSS | HLA-DRB1*12:01, HLA-DRB1*01:01, HLA-DQA1*01:02/DQB1*06:02, HLA-DQA1*05:01/DQB1*03:01 |
|  | LEDRLVETL | HLA-B*40:01 | GARAGAPGRVSFYPA | HLA-DQA1*05:01/DQB1*03:01 |
|  | QTPFGAGWSW | HLA-B*57:01, HLA-B*58:01, HLA-B*53:01, HLA-A*32:01 | PGARAGAPGRVSFYP | HLA-DQA1*05:01/DQB1*03:01 |
|  |  |  | AFAAPISALNYAFTP | HLA-DRB1*12:01, HLA-DRB1*04:01, HLA-DQA1*05:01/DQB1*03:01, HLA-DRB1*15:01 |
| **2** | FQFALANAF | HLA-A*02:06, HLA-B*39:01, HLA-B*15:01, HLA-B*35:01, HLA-C*12:03, HLA-B*15:02, HLA-C*14:02 | EKRFQVHEPNISAWR | HLA-DRB3*02:02, HLA-DRB1*04:01, HLA-DRB1*09:01, HLA-DRB1*01:01, HLA-DRB1*07:01, HLA-DRB1*04:05, HLA-DRB5*01:01 |
|  | FTIPLPGDR | HLA-A*68:01 | AGFRQYRAASIQVGN | HLA-DRB1*15:01, HLA-DRB1*04:05, HLA-DRB1*07:01, HLA-DQA1*05:01/DQB1*03:01, HLA-DRB1*09:01, HLA-DQA1*01:02/DQB1*06:02, HLA-DRB3*02:02, HLA-DRB1*04:01, HLA-DRB1*08:02, HLA-DPA1*02:01/DPB1*14:01, HLA-DRB1*01:01, HLA-DRB3*01:01, HLA-DQA1*01:01/DQB1*05:01 |
|  | SAADVAVVV | HLA-C*12:03, HLA-A*68:02, HLA-A*02:06, HLA-A*68:01, HLA-C*03:03 | HELPFQFALANAFTL | HLA-DRB1*09:01, HLA-DRB1*01:01, HLA-DRB1*07:01, HLA-DRB5*01:01, HLA-DRB1*04:05, HLA-DRB3*01:01, HLA-DRB1*04:01, HLA-DRB4*01:01, HLA-DPA1*01:03/DPB1*02:01, HLA-DRB1*13:02, HLA-DRB3*02:02 |
| 3 | SSNVNFPLY | HLA-A*01:01, HLA-A*30:02, HLA-B*57:01, HLA-B*15:01, HLA-A*11:01, HLA-B*35:01, HLA-A*26:01, HLA-A*30:01 | SLNLLSLILISSNVN | HLA-DRB1*04:03, HLA-DRB1*04:05, HLA-DRB1*04:01, HLA-DRB1*04:04, |
|  | CLIGTAPNV | HLA-A*02:01, HLA-A*02:03, HLA-A*02:06, HLA-A*68:02, HLA-B*51:01 | LSLILISSNVNFPLY | HLA-DRB1*04:01, HLA-DRB3*03:01, HLA-DRB1*13:02, HLA-DRB1*04:04, HLA-DRB1*04:05 |
|  | TTNGTVTLP | HLA-A*68:02, HLA-A*30:01, HLA-A*30:02, HLA-A*26:01, HLA-A*01:01, HLA-A*68:01, HLA-B*58:01 |  |  |
| 4 | QTYGAKIAR | HLA-A*68:01, HLA-A*31:01, HLA-A*33:01, HLA-A*11:01, HLA-A*03:01, HLA-A*30:01, HLA-A*26:01 | NVGANAFLSGTRPRL | HLA-DRB5*01:01, HLA-DQA1*05:01/DQB1*03:01, HLA-DRB3*02:02, HLA-DRB1*09:01, HLA-DRB1*04:01 |
|  | SEYVWNYEL | HLA-B*40:01, HLA-B*44:03, HLA-B*44:02, HLA-A*02:06, HLA-B*08:01, HLA-A*32:01, HLA-B*51:01 | NAFLSGTRPRLNLSL | HLA-DRB3*02:02, HLA-DRB5*01:01, HLA-DRB1*04:01, HLA-DRB1*07:01, HLA-DRB1*01:01, HLA-DRB1*09:01 |
|  | RYFHGTQDEF | HLA-A*24:02, HLA-A*23:01, HLA-A*30:02, HLA-A*32:01, HLA-B*15:01 | ARAPAYTANMGAKYQ | HLA-DRB3*02:02, HLA-DRB3*01:01, HLA-DRB1*09:01, HLA-DRB1*13:02, HLA-DRB5*01:01, HLA-DRB1*15:01 |
|  |  |  | AKYQFLKGWELSSNV | HLA-DRB1*01:01, HLA-DRB1*15:01, HLA-DRB1*04:05, HLA-DRB5*01:01, HLA-DRB1*04:01, HLA-DRB1*07:01 |

Table S2: Physiochemical Properties, Antigenicity and Allergenicity of the Vaccine

| **Property** | **Measurement** | **Indication** |
| --- | --- | --- |
| Total Number of Amino Acid | 747 | Appropriate |
| Molecular Weight | 78.22386 KDa | Appropriate |
| Formula | C_3492_H_5396_N_1006_O_1031_S_8_ | - |
| Theoretical pI | 9.72 | Basic |
| Total number of positively charged residues (Arg + Lys) | 54 | - |
| Total number of negatively charged residues (Asp + Glu | 81 | - |
| Total Number of Atoms | 10933 | - |
| Instability index (II) | 33.01 | Stable |
| Aliphatic Index | 66.44 | Thermostable |
| Grand Average of Hydropathicity (GRAVY) | -0.419 | Hydrophilic |
| Antigenicity VaxiJen | 1.0250 | Antigenic |
| Antigenicity AntigenPro | 0.831943 | Antigenic |
| Allergenicity | Non-allergenic | Non-allergenic |
| Toxicity | Non-toxic | Non-toxic |
